# Visual Exploration during Locomotion Limited by Fear of Heights

**DOI:** 10.1371/journal.pone.0105906

**Published:** 2014-08-28

**Authors:** Günter Kugler, Doreen Huppert, Maria Eckl, Erich Schneider, Thomas Brandt

**Affiliations:** 1 Institute of Clinical Neurosciences, University of Munich, Munich, Germany; 2 German Center for Vertigo and Balance Disorders, University of Munich, Munich, Germany; 3 Brandenburg University of Technology Cottbus – Senftenberg, Senftenberg, Germany; University of Muenster, Germany

## Abstract

**Background:**

Visual exploration of the surroundings during locomotion at heights has not yet been investigated in subjects suffering from fear of heights.

**Methods:**

Eye and head movements were recorded separately in 16 subjects susceptible to fear of heights and in 16 non-susceptible controls while walking on an emergency escape balcony 20 meters above ground level. Participants wore mobile infrared eye-tracking goggles with a head-fixed scene camera and integrated 6-degrees-of-freedom inertial sensors for recording head movements. Video recordings of the subjects were simultaneously made to correlate gaze and gait behavior.

**Results:**

Susceptibles exhibited a limited visual exploration of the surroundings, particularly the depth. Head movements were significantly reduced in all three planes (yaw, pitch, and roll) with less vertical head oscillations, whereas total eye movements (saccade amplitudes, frequencies, fixation durations) did not differ from those of controls. However, there was an anisotropy, with a preference for the vertical as opposed to the horizontal direction of saccades. Comparison of eye and head movement histograms and the resulting gaze-in-space revealed a smaller total area of visual exploration, which was mainly directed straight ahead and covered vertically an area from the horizon to the ground in front of the feet. This gaze behavior was associated with a slow, cautious gait.

**Conclusions:**

The visual exploration of the surroundings by susceptibles to fear of heights differs during locomotion at heights from the earlier investigated behavior of standing still and looking from a balcony. During locomotion, anisotropy of gaze-in-space shows a preference for the vertical as opposed to the horizontal direction during stance. Avoiding looking into the abyss may reduce anxiety in both conditions; exploration of the “vertical strip” in the heading direction is beneficial for visual control of balance and avoidance of obstacles during locomotion.

## Introduction

Looking into an abyss is the precipitating stimulus that induces fear of heights in susceptible individuals. This distressing experience is associated with individually varying amounts of anxiety, inner agitation, a queasy-stomach feeling, unsteadiness, dizziness, impairment of gait, and weakness of the knees [1]. Usually the threatening stimulus is avoided.

Our first study on visual exploration of the surroundings by susceptible subjects while standing still and looking from a balcony 20 meters above ground revealed that spontaneous eye and head movements were significantly diminished. Gaze-in-space was also restricted, in particular fearful subjects preferred to direct their gaze to the horizon [2]. This was interpreted to be a strategy for alleviating fear of heights, since the horizontal distance to remote visual targets is not as threatening as depth is. Similarly earlier studies on specific phobias showed that fearful subjects tend to avoid gazing towards the threat [3], [4], and fearful subjects tend to overestimate height [5].

The question arose as to whether visual exploration is similarly reduced during locomotion at heights, which differs from standing still in several aspects. Self-motion has been shown to increase anxiety in patients with acrophobia during real or virtual stimulation [6]. It is well acknowledged that anxiety not only modulates postural control and locomotion [7] but also gaze and ocular motor control [8]. One of the major findings of the laboratory study of Tersteeg et al. was that knowledge about the increased possibility of falling is decisive for adapting gait in an exposed situation [9]. Furthermore, locomotion requires visual adjustment of the direction of self-motion and detection of obstacles at least two steps ahead of foot placement [10]. Visual feedback is particularly relevant for the lateral stabilization of gait [11]. Consequently, a limited gaze behavior might increase the risk of falling. Likewise visual conditions that lead to asymmetrical visual flow might also be expected to influence locomotion. Individuals with a fear of heights have been shown to be affected more strongly than controls in virtual visual flow stimulation [12], by exhibiting stronger body sway and reporting higher anxiety and dizziness.

In the current study movements of the eyes and the head were recorded separately in subjects susceptible to fear of heights and in non-susceptible controls while walking on an emergency escape balcony projecting from the fourth floor of a building. Video recordings of the subjects were simultaneously made to individually correlate gaze and gait behavior. In view of our previous findings on upright stance, we expected that visual exploration behavior, both eye and head movements, would diminish. We further hypothesized that the gait as well as the heading direction in fearful subjects would be affected. A major question was whether subjects susceptible to fear of heights exhibit a common visual exploration pattern or if compensatory strategies are chosen individually.

## Methods

### Subjects

Sixteen subjects (11 females, aged 29 to 72, mean 47.7) with self-reported fear of heights were assessed with a detailed questionnaire [1] before participating in the locomotion experiment. They fell into the category of visual height intolerance [13], having reported fear of heights on several previous occasions. These subjects were called “susceptibles” (to fear of heights). In addition, 16 subjects (9 females, aged 25 to 72, mean 48.3) without fear of heights served as the control group. All subjects had participated in a previous experiment on visual exploration at heights during upright stance [2]. No subject reported psychiatric, neurologic, vestibular, or balance disorders.

**Figure 1:**
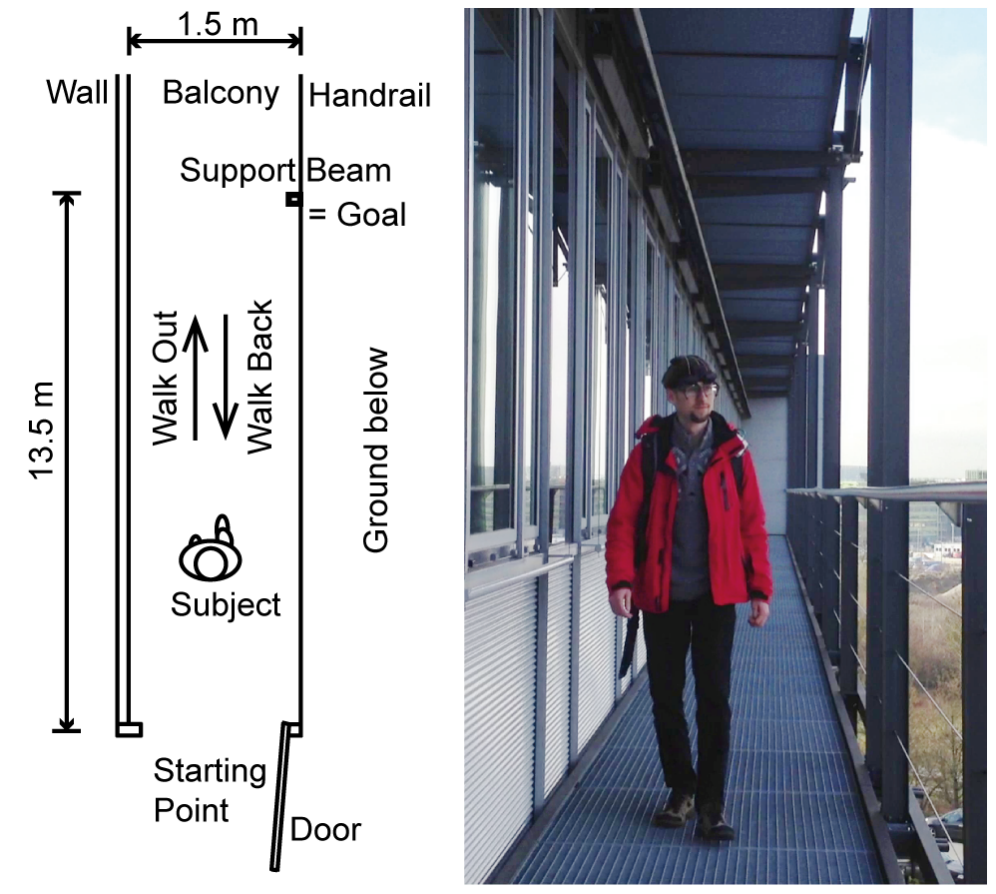
Experimental setup. Subject walks on the grid floor of an escape balcony until he/she reaches the target (a support beam of the balcony), then turns and walks back. Subject wears mobile eye-tracking goggles with a head-fixed scene camera, and 6-degrees-of-freedom inertial sensors.

Subjects gave their informed written consent prior to participation. The study was conducted in accordance with the Declaration of Helsinki and was approved by the Ethics Committee of the University of Munich Hospital. The individual depicted in fig. 1 has given written informed consent to the publication of this figure.

### Experimental Setup and Procedure

An emergency escape balcony with a metallic floor grid, about 20 meters above ground level, was used as the site for exposure to height. The balcony had a handrail for safety, but the view remained nearly unobstructed (fig. 1).

Before beginning the locomotion experiment, subjects were equipped with a mobile infrared video eye-tracking system, consisting of goggles, a head-fixed camera, and a backpack with a recording laptop. The goggles had 6-degrees-of-freedom inertial sensors integrated for measurements of head movements in the three planes yaw, pitch, and roll. The subjects were given time to familiarize themselves with the equipment in a hallway inside the building but were not allowed to look outside and estimate the height of the site of the experiment.

The eye-tracker recorded binocular eye movements in two dimensions (horizontal and vertical) at a frame rate of 120 Hz. Eye position in head was projected onto the recording of the head-fixed scene camera, which recorded at a frame rate of 25 Hz. Eye movements were calibrated with a 5-point calibration protocol. The calibration dots were projected from a laser unit attached to the head-fixed camera. In addition, the walking subjects were recorded by an external video camera.

The experimental protocol consisted of a locomotion task that was performed twice to assess possible habituation. Subjects were instructed to walk out onto the balcony to a designated goal (a support beam of the balcony which stood 13.5 m from the door, fig. 1), then turn around and come back. Each of the two locomotion tasks was split into two parts for the analysis: “walk out” from leaving the door until reaching the goal and “walk back” starting after the turn until reaching the door. After each task subjects were asked to rate their feelings of fear on a subjective scale of 0 to 10 (0 = no fear, 10 = maximal). Subjects in the susceptible group, reporting no fear at all (n = 2; 0 on the scale, both female) during both walks were excluded from analysis. One male subject was not able to walk the whole distance out onto the balcony due to excessive anxiety and was therefore excluded. The data of the remaining 13 participants underwent further analysis. No subject in the control group reported any fear in either walk. One control subject (female) was excluded from statistical analysis of the head and eye movements, and one susceptible subject (female) was excluded from statistical analysis of gait parameters. Both were classified as outliers; details are given in the respective paragraphs.

Time from start to stop was recorded for all walks. The turn itself was omitted from analysis and results. At the end of the experiment, participants were interviewed about their body symptoms and coping strategies.

### Head Movement Analysis

The data from the inertial measurement unit was processed with the following procedures to obtain the mean absolute velocities in the three planes and the mean total velocity. Raw angular velocity data from the inertial measurement unit was filtered using a Butterworth band-pass filter with cutoff frequencies of 0.5 Hz and 25 Hz. The mean absolute velocity was calculated by averaging the absolute velocity signal. The mean absolute velocities for yaw, pitch, and roll planes were obtained by averaging three sensor channels individually. A principal component analysis was performed for the head movements in the three planes in combination with the absolute head movements to determine the correlations between the dimensions.

The velocities in yaw and pitch were aggregated in a 2-dimensional histogram for each group with bin size 1°/s × 1°/s. The histograms of each individual were normalized to the duration prior to groupwise aggregation, to ensure the equal contribution of each individual to the cumulative histogram. The difference between the groups was calculated by subtracting the values of the control group from the susceptible group for each bin in the 2-d histogram.

Amplitudes of head oscillations were determined for each subject as follows. The absolute signal of the inertial acceleration data was integrated twice, after subtraction of the mean of the data to eliminate gravity acceleration from the signal. During steady-state locomotion (from 2 s after begin of each walk until 2 s before end of walk), the resulting positional data reflect vertical head oscillations.

The head orientation was calculated by non-commutative 3-dimensional integration of the Butterworth-filtered angular velocity. The reference position for the head orientation was taken from the head-fixed camera recordings. The reference orientation was chosen so that it pointed to the middle of the horizontal orientation of the balcony in the direction of walking. The median head orientation and interquartile range were calculated for the yaw and pitch planes for each subject and each walk.

### Eye Movement Analysis

Eye movements were submitted to an iterative algorithm using velocity and acceleration criteria [14] to identify fast phases (saccades) and slow phases (fixations, vestibulo-ocular reflex movements). Fast phase entry and exit points were defined to occur at 10% of the maximal velocity of the corresponding saccade. The maximal velocities and amplitudes of saccades were calculated as well as the durations of the slow phases. The left eye was selected as the standard for analysis. However, the data of the right eye were selected manually if they were less affected by noise resulting from infrared environmental illumination and the different lighting conditions before and after the turn on the balcony. Segments for which both eyes did not provide sufficient data quality were excluded manually from analysis. Histograms of the directions of all identified saccades were calculated for each group.

Gaze-in-space directions were determined by combining head orientation and eye-in-head orientation. “Heatmaps” for both groups were calculated to indicate the gaze directions during walking. For each fixation, a circular shape extending 5° in diameter (roughly corresponding to the fovea) was attributed in order to represent the direction of gaze during this fixation in the cumulative heatmaps.

### Gait Analysis

The number of steps was derived from the linear acceleration obtained by the inertial measurement unit. In three cases in which steps could not be identified automatically, they were obtained manually by evaluating the external video recordings. Mean step length was calculated by dividing the number of steps by the walking distance; the mean step frequency was calculated by dividing the number of steps by the duration of the corresponding walk; and the mean walking velocity was calculated by dividing the walking distance by duration. Each participant’s lateral distance from the wall was obtained from the video recordings at a fixed point during the first walk. The distance was measured from the wall to the first contact of the left foot after the subject had walked 3 m on the balcony.

Two experienced neurological clinicians (DH, TB) who were absent during the video recording of the walks assigned the subjects to two groups (fearful and not fearful), after watching the external video recording of the locomotion. No criteria for assignment had been given. After the experiment, the neurologists were questioned about the criteria they had used as the basis for their evaluations.

### Statistical Analysis

The data were analyzed and the confidence intervals calculated with MATLAB, Mathworks Inc. Version 2010a. Statistical tests were calculated in SPSS version 21. A multivariate general linear model was calculated with the four main dependent variables *mean head velocity*, *saccade amplitudes*, *saccade frequencies*, and *gait velocity*, with the three independent variables *group*, *direction* and *repetition* of the walk, while controlling for *age* and *gender*. To evaluate the significant findings and identify individual contributions, follow-up univariate mixed-model ANOVAs were performed for individual variables. In addition, a multivariate general linear model was calculated with the head orientation parameters *yaw range, pitch range*, *yaw median position, and pitch median position* as dependent variables and the same independent variables *group*, *direction* and *repetition* of the walk, while controlling for *age* and *gender*. Correlation analysis was performed with R version 3.0.2 [15]. One-sided significance tests were performed for Pearson product-moment correlation coefficients.

## Results

The descriptive statistics of eye and head movements and gait parameters are given in Table 1. There the main variables investigated, i.e., mean head movement velocity, saccade amplitudes and frequencies, and gait velocity, are reported. Additional parameters, i.e., head movement range, median heading direction, fixation durations, and step length and frequency, are given to fully illustrate the behavior of susceptibles and controls. The multivariate general linear model for the main variables revealed main effects of group (p<.001, using Pillai’s trace V = .653, F(4,22) = 10.34), direction (p<.001, using Pillai’s trace V = .761, F(4,22,) = 17.47), and repetition (p = .004, using Pillai’s trace V = .490, F(4,22) = 5.29). The interaction term group × direction showed a trend (p = .051, using Pillai’s trace V = .337, F(4,22) = 2.80). None of the other interactions were significant. Follow-up univariate ANOVAs are reported in the corresponding sections. Table 2 depicts the correlation analysis.

**Table 1:** Descriptive statistics.

		Walk 1				Walk 2			
		Out		Back		Out		Back	
		SUSC	CONTR	SUSC	CONTR	SUSC	CONTR	SUSC	CONTR
**Head**									
Mean Head Velocity [°/s]		14.6±5.1	19.8±5.1	16.6±6.0	21.3±4.3	12.2±3.7	18.9±4.6	15.0±5.2	20.9±4.3
Median Head Direction, Yaw [°]		5.0±5.6	8.4±10.2	3.5±14.8	12.0±12.0	4.3±5.2	11.2±11.9	3.8±14.5	11.0±12.7
Head Interquartile Range, Yaw [°]		6.6±5.6	14.5±12.1	7.8±5.3	21.2±25.8	5.2±3.6	17.8±16.0	6.8±4.6	16.6±14.9
Median Head Direction, Pitch [°]		**−**2.0±4.3	1.7±4.2	**−**3.6±3.2	**−**4.6±6.9	**−**2.6±5.7	**−**0.7±6.6	**−**0.6±2.3	**−**4.1±7.8
Head Interquartile Range, Pitch [°]		4.9±4.7	7.4±4.4	6.7±5.8	10.9±7.4	4.8±3.8	10.2±9.1	4.4±2.4	9.7±6.0
**Eye**									
Saccade Amplitude [°]		11.4±3.3	10.8±3.87	10.8±3.1	12.5±4.8	10.2±1.9	9.0±2.31	11.0±4.1	10.8±2.1
Saccade Frequency [1/s]		2.93±0.93	3.17±0.72	2.87±0.88	3.11±0.63	2.83±0.85	3.13±0.7	2.99±0.73	3.01±0.78
Fixation Duration [s]		0.32±0.16	0.28±0.11	0.33±0.15	0.27±0.09	0.36±0.21	0.28±0.09	0.30±0.10	0.30±0.14
**Gait**									
Velocity [m/s]		0.79±0.22	1.14±0.18	0.96±0.21	1.19±0.17	0.91±0.2	1.16±0.2	1.05±0.15	1.23±0.18
Step Frequency [1/s]		1.41±0.21	1.76±0.19	1.55±0.19	1.78±0.2	1.51±0.19	1.76±0.25	1.57±0.15	1.79±0.2
Step length [m]		0.54±0.1	0.63±0.07	0.61±0.08	0.65±0.07	0.58±0.08	0.64±0.08	0.64±0.07	0.66±0.07

Head movement, eye movement, and gait parameters. Mean values for group are given with standard deviations.

**Table 2:** Correlation results.

		Walk 1		Walk 2	
		Out	Back	Out	Back
**Head**					
Mean Head Velocity		**r = −0.50 p = 0.040**	**r = −0.60 p = 0.015**	**r = −0.56 p = 0.023**	**r = −0.49 p = 0.043**
Head Interquartile Range Yaw		r = **−**0.32 p = 0.16	**r = −0.58 p = 0.023**	**r = −0.63 p = 0.014**	**r = −0.60 p = 0.020**
Head Interquartile Range Pitch		**r = −0.60 p = 0.019**	r = **−**0.48 p = 0.059	**r = −0.50 p = 0.049**	r = **−**0.29 p = 0.18
**Eye**					
Saccade Amplitudes		**r = −0.50 p = 0.040**	r = **−**0.41 p = 0.080	r = **−**0.35 p = 0.12	r = **−**0.40 p = 0.090
Saccade Frequencies		r = **−**0.41 p = 0.080	r = **−**0.25 p = 0.20	r = **−**0.46 p = 0.057	r = **−**0.33 p = 0.14
**Gait**					
Velocity		r = **−**0.44 p = 0.075	**r = −0.52 p = 0.040**	**r = −0.57 p = 0.026**	r = 0.07 p = 0.58

Correlations of determined parameters with subjective fear of the susceptibles. Pearson’s r and p-values are given; significant results are marked in bold (p<0.05).

### Subjects

When asked to rate their subjective fear, susceptibles reported values ranging from 0 to 8 (median of 4) during the first walk out and back, and values in the range of 0.5 to 8.5 (median of 3) during the second walk. The mean value decreased non-significantly by 0.23 from the first to the second walk (t-test, p = 0.50). None of the controls reported any fear during the experiments.

The body symptoms indicated were inner agitation (reported by 69%), subjective postural imbalance (69%), queasy stomach feeling (62%), anxiety (62%), weakness in the knees (54%), sweating (38%), palpitation (31%), dizziness (23%), lightheadedness (8%), and trembling (8%). No susceptible subject was entirely symptom-free during exposure.

Reported coping strategies were staying close to the wall of the building (54%), avoiding looking down into the depths (46%), thinking about gripping the handrail (38%), conscious control of breathing (38%), and thinking about dropping out of the experiment (15%).

### Head Movements

**Figure 2:**
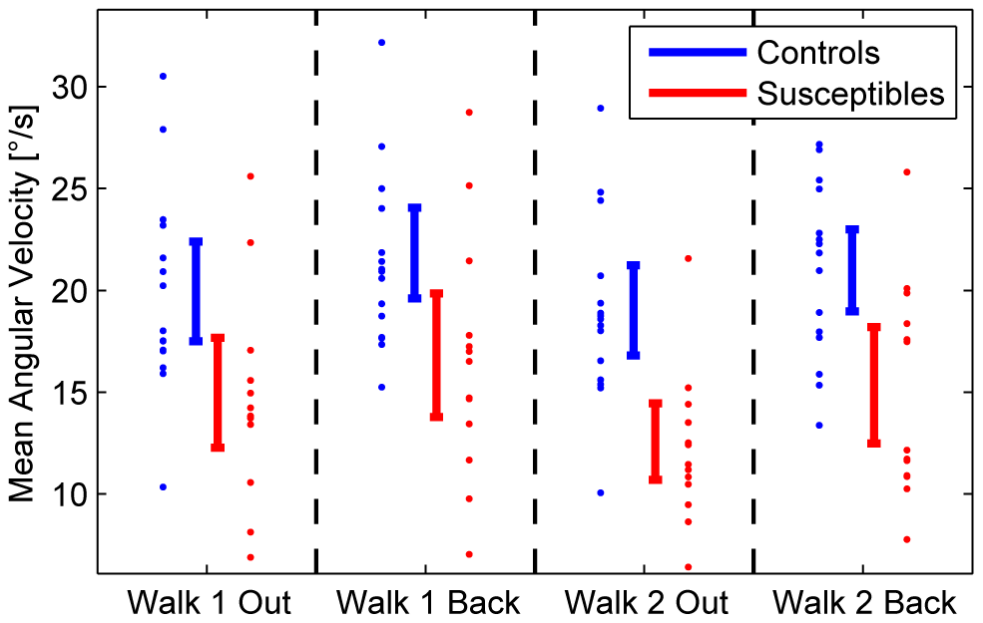
Head Movements. Total head movements (in the yaw, pitch, and roll planes) averaged over time (mean angular velocities) for susceptibles (red) and controls (blue). Vertical bars denote the confidence intervals for the means during locomotion over a distance of 13.5 m on the emergency escape balcony. Depicted are all four trials: Walk 1 out and back and Walk 2 out and back. Susceptibles perform significantly fewer head movements as a group (p = .002). A comparison of the two conditions walking out and back revealed fewer head movements when walking out (p = .001).

The follow-up univariate ANOVA for normalized head movements showed significant effects of group (F(1,25) = 11.66, p = .002), repetition (F(1,25) = 4.96, p = .035), and direction (F(1,25) = 14.89, p = .001). Normalized head movements were reduced in susceptibles compared to controls (fig. 2). A comparison of the walks out and back showed that head movements were smaller during the walk out than during the walk back on the balcony.

Normalized movements in the yaw plane were largest in both groups (8.9°/s in susceptibles vs. 12.4°/s in controls), followed by pitch movements (7.6°/s vs. 9.2°/s), and roll movements (5.2°/s vs. 7.5°/s). A principal component analysis revealed that the single dimensions were correlated (the first principal component explains 80% of total variance). However, pitch movements were less reduced (to 83% of controls’ mean pitch movement) than yaw (72%) and roll movements (69%). Data from one control subject were considered outliers due to the subject’s extremely large exploratory movements (mean value 49°/s in the four walks compared to 20°/s for the control group, i.e., corresponding to 6 standard deviations above the group mean) and were thus excluded from head and eye movement statistics.

**Figure 3:**
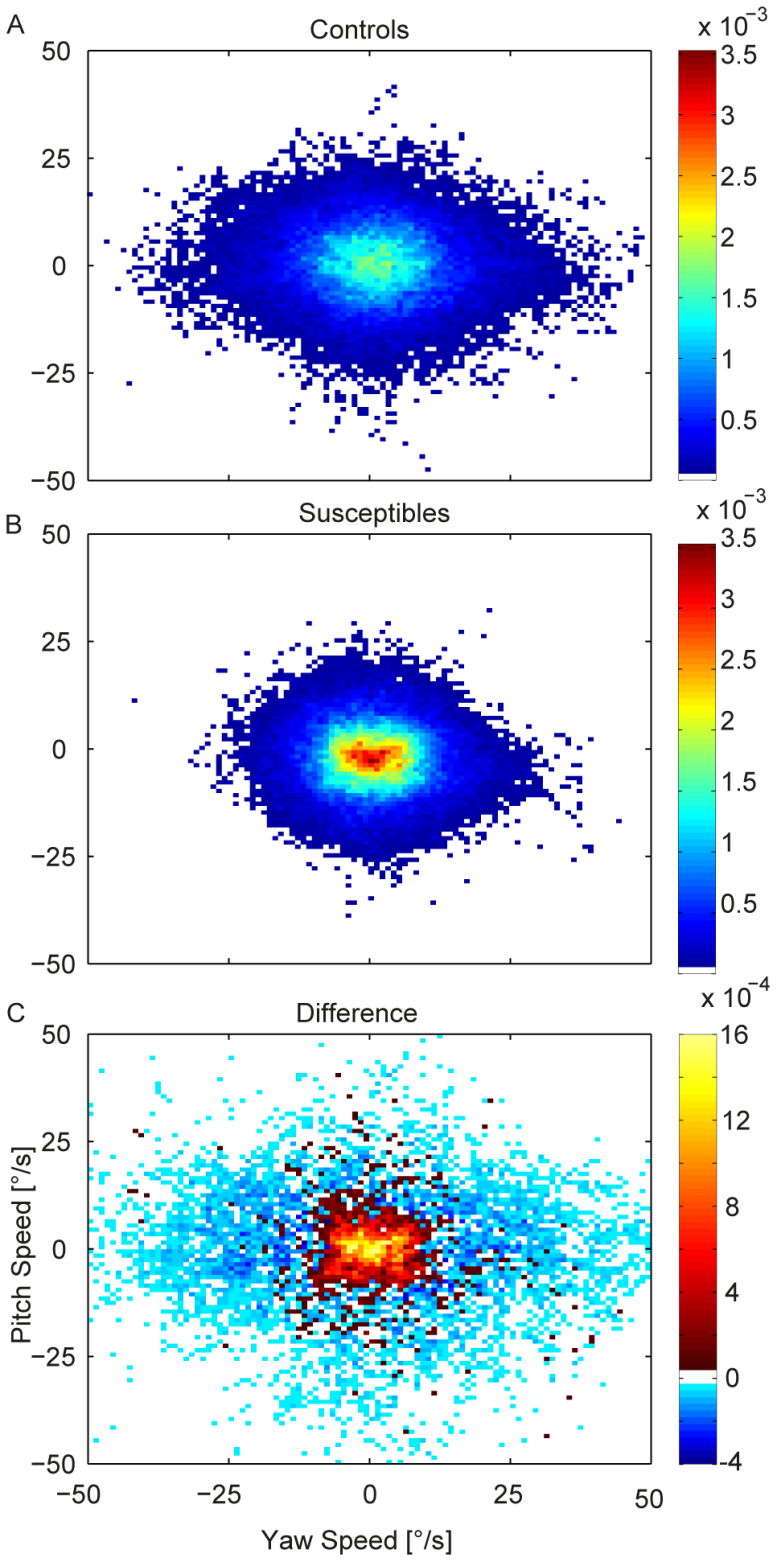
Head velocity histograms. Histograms of head movement velocities in yaw and pitch for all walks in controls (A) and susceptibles (B). Yaw plane is mirrored for the walks back. Colors show the normalized frequencies of pairs of yaw and pitch velocities. A comparison is depicted in C, hot colors indicate that susceptibles exhibit more corresponding head velocities than controls; cold colors, that controls exhibit more corresponding velocities than susceptibles. The difference plot reveals that susceptibles exhibit less fast head velocities.

**Figure 4:**
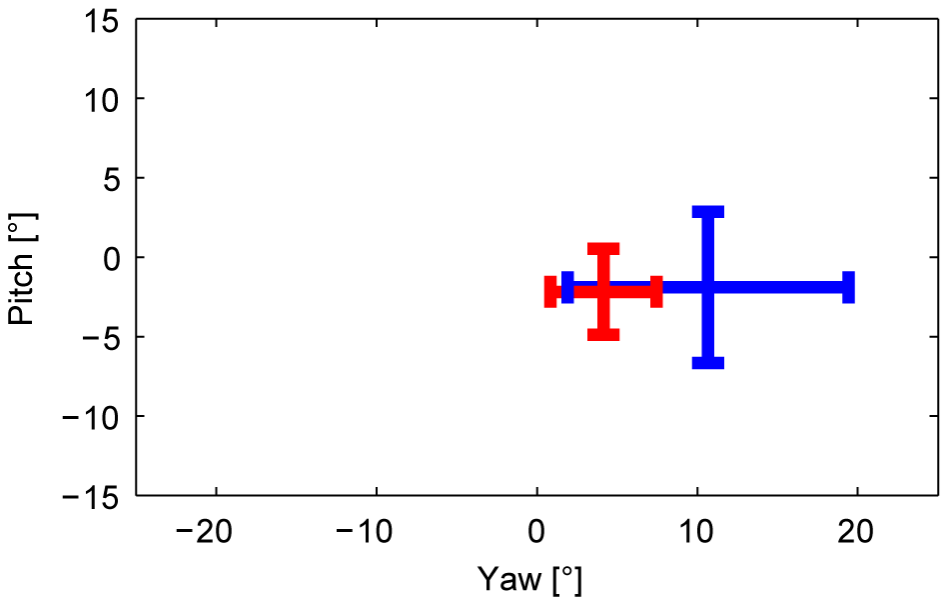
Head Position. Median head positions while walking on the balcony in the horizontal (yaw) and vertical planes (pitch) for susceptibles (red) and controls (blue), pooled for all four walking conditions. Abscissa indicates deviations of horizontal head position from balcony midline in degrees (0° reference); ordinate indicates deviations of vertical head position from earth horizontal in degrees (0°). Depicted are the group mean interquartile ranges (bars), centered at the mean group median head positions in both planes (crossing). Horizontal head positions for the walk back conditions are mirrored so that all positive values reflect head positions toward the open side opposite the wall (depth), and negative values represent head orientations toward the wall side. For the vertical plane, positive values indicate head extension, negative head flexion. Susceptibles direct their head less toward the open side (yaw median: p = .062), and restrict it to a smaller area (yaw range: p = .013; pitch range: p = .034).

Calculations of translational head movements showed smaller translational oscillations in the susceptible group (23 mm amplitudes vs. 36 mm amplitudes in controls; t-Test, p<0.001), particularly along the vertical axis. Histograms of head movement angular velocities (fig. 3) in yaw and pitch showed that susceptibles performed fewer fast head movements than controls. Controls showed an anisotropy with higher velocities in the yaw plane, which was much less pronounced in susceptibles. After the significant outcome of the head movement velocity test, a subsequent multivariate general linear model was calculated for the head orientation and direction parameters. The general linear model revealed a main group effect (p = .016, using Pillai’s trace V = .411, F(4,22) = 3.84), while neither the main repeated factors (direction, repetition) nor any of the interactions were significant. Follow-up univariate tests revealed significant outcomes in the factor group for head movement range (yaw range F(1,25) = 7.11, p = .013 and pitch range F(1,25) = 5.02, p = .034). The interquartile ranges of the head positions in yaw and pitch were smaller in susceptibles; controls explored a wider area (fig. 4). Pitch median direction did not show a significant group effect (F(1,25) = .062, p = .80), while yaw median direction revealed a trend (F(1,25) = 3.83, p = .062). The median head direction of susceptibles in the horizontal plane was mainly straight ahead. In contrast, controls preferred the open side of the balcony opposite the wall.

Correlation analysis showed moderate to strong correlations of mean head velocities and interquartile ranges of head orientation with the subjective fear of susceptibles (table 2).

### Eye Movements

The follow-up univariate ANOVAs for eye movements did not show a significant group effect (saccade amplitudes: F(1,25) = .23, p = .63 and saccade frequencies: F(1,25) = .28, p = .60). Saccade amplitudes showed a tendency to be smaller during the second walk on the balcony (factor repetition: F(1,25) = 4.25, p = 0.05). Correlation analysis showed moderate, but mostly nonsignificant correlations of eye movement parameters with the subjective fear of susceptibles.

**Figure 5:**
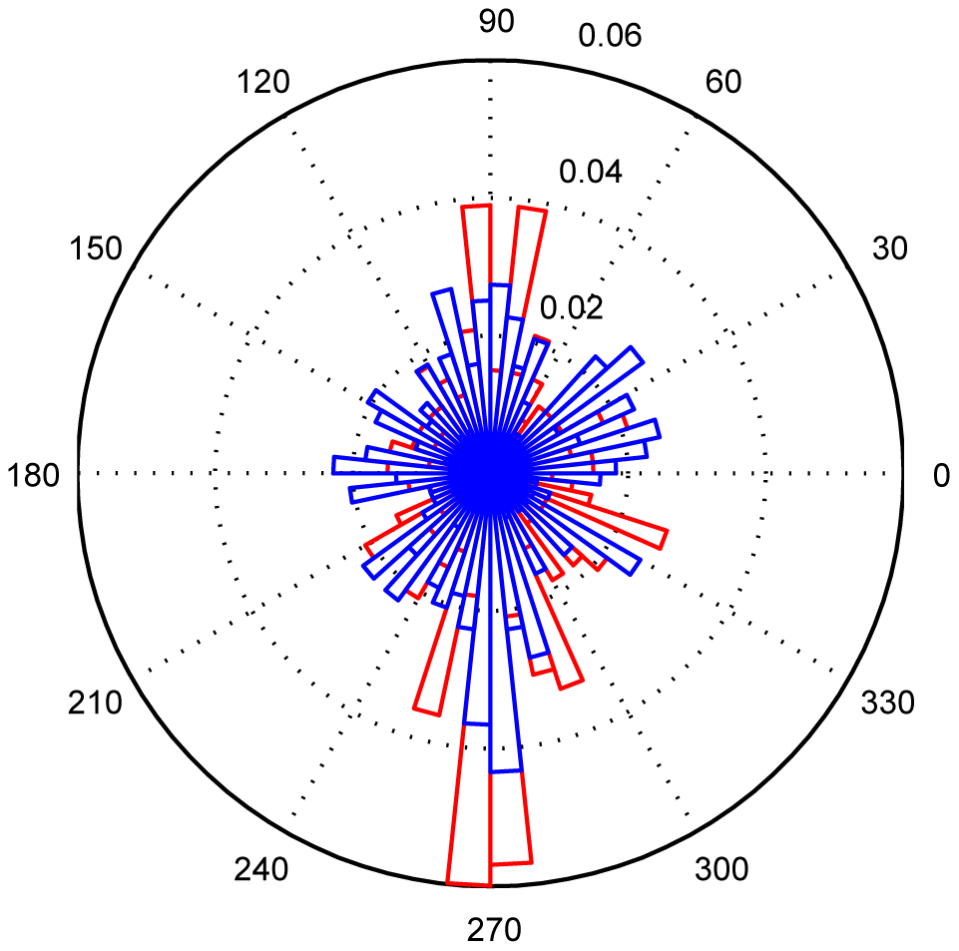
Saccade direction histograms. Histogram of the directions of eye-in-head movements for susceptibles (red) and controls (blue) during locomotion (all four walks). Movements are depicted in degrees (0° = horizontal movement to the left, 180° = horizontal to the right, 90° = vertical up, 270° = vertical down). Dotted circles (0.01–0.06) represent percentage of eye movements performed in angular ranges of 6°. Susceptibles perform more eye movements in the vertical direction, and fewer in the horizontal direction compared to controls.

The histograms of saccade directions (fig. 5) yielded two results. First, both groups performed more saccades vertically than horizontally. Second, the anisotropy was slightly more pronounced in susceptibles than in controls.

### Gaze-in-space

**Figure 6:**
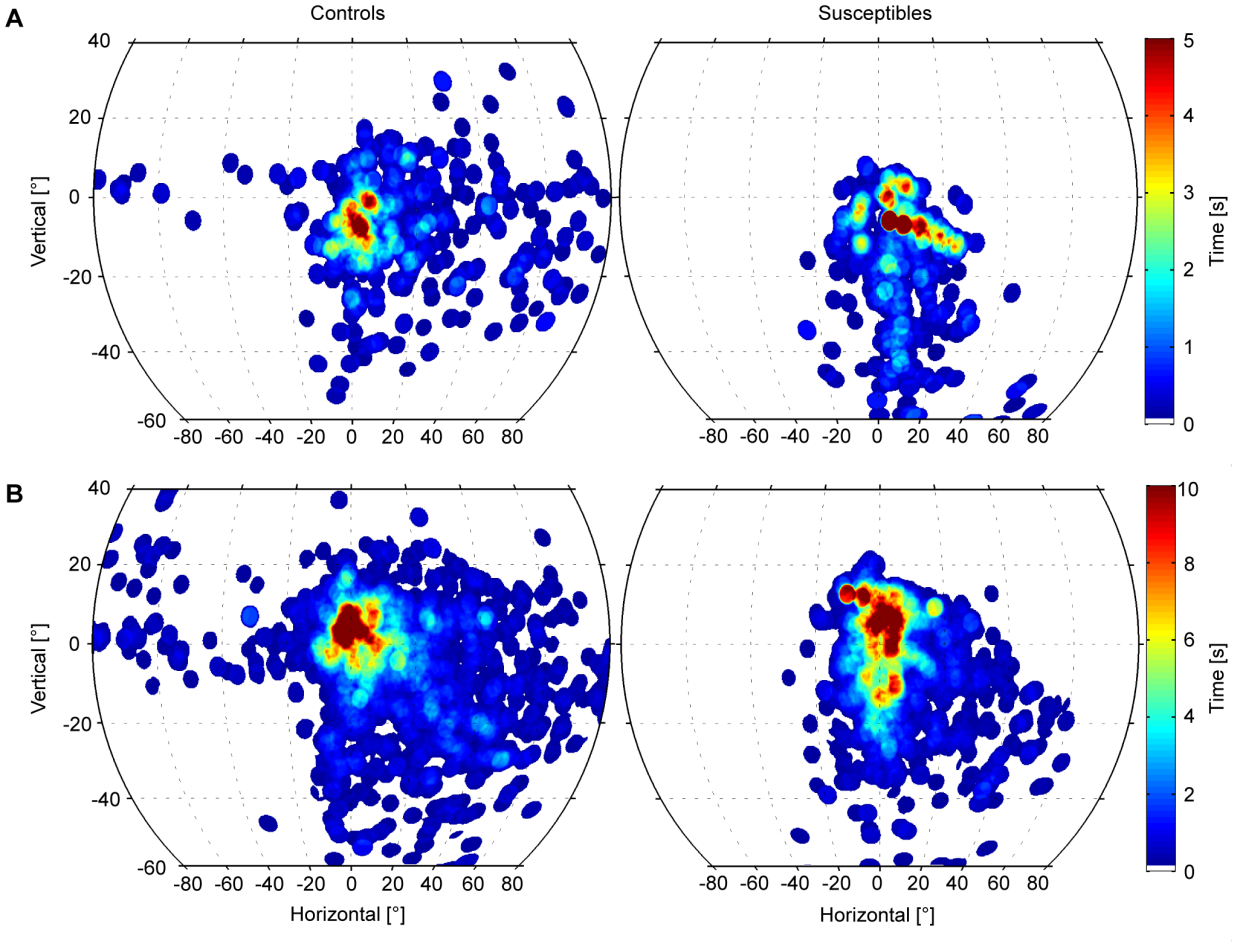
Gaze in Space. Fixations of environmental structures with combined eye and head movements, during locomotion, for controls (left) and susceptibles (right). The number of subjects (coded by color) fixating identical targets within an area extending horizontally 160°, vertically 100° of the body-centered surroundings (0° ordinate = horizon, 0° abscissa = straight ahead) is depicted. Data are shown in Mollweide equal area projection. A shows data for Walk 1 Out, B shows cumulative data for all four walks, with mirrored horizontal coordinates for the walks back. Explored areas of the controls tend to cover the entire surround towards the open side of the balcony (depth). Susceptibles direct their gaze less to the open side than controls, and more directly ahead to the goal, the floor, and the handrail.

Heatmaps of gaze-in-space distributions are depicted in fig. 6. Both groups spent an essential amount of time gazing towards the goal of movement (the support beam during the walk out and the door on the way back). While the control group freely explored the open side and depth, the susceptibles restricted their gaze mainly to straight ahead, the floor, and the handrail. This behavior was especially prominent in the first walk out (fig. 6A), but persisted in all walks (fig. 6B).

### Gait Analysis

The experienced clinicians who assigned the participants to one of two groups on the basis of the video-recorded walks identified 27 participants correctly, assigned 1 participant to the wrong group (false negative), and one clinician assigned 1 participant correctly while the other assigned the same participant incorrectly (false positive). The criteria used by the neurologists included slowing of gait, less swinging of the arms, smaller and cautious steps, and less vertical head and body oscillations during the gait cycle.

**Figure 7:**
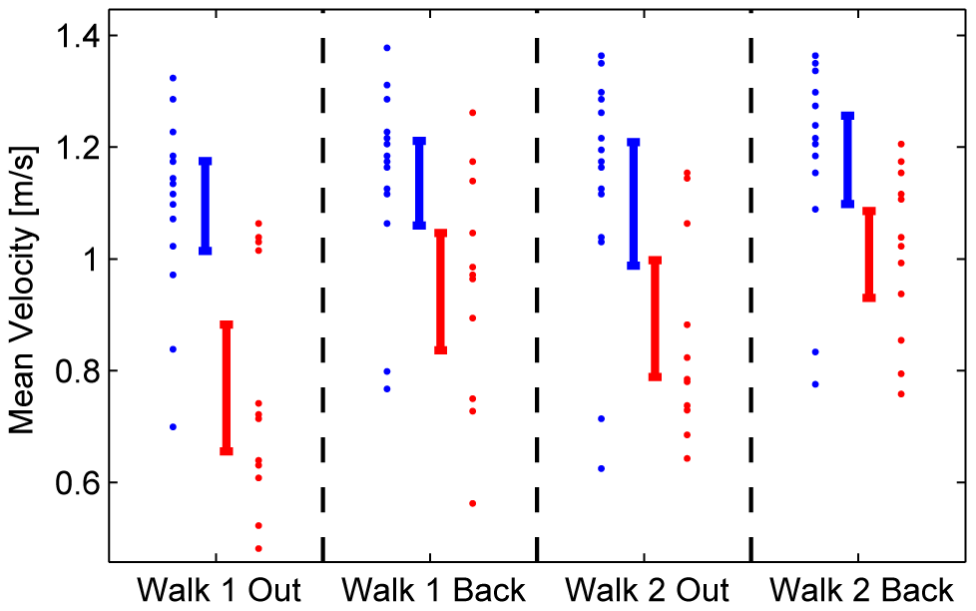
Gait velocity. Gait velocity (m/s) in all four walks for susceptibles (red) and controls (blue). Vertical bars denote the confidence intervals for the means during locomotion over a distance of 13.5 m on the balcony. Susceptibles walk slower than controls (p = .001). On the way back, susceptibles speed up; this relative increase in speed was revealed by the marginally significant interaction (p = .07).

The univariate follow-up ANOVA revealed that susceptibles exhibited a slower mean locomotion speed (significant group effect: F = 13.59, p = .001), related to smaller mean step frequencies and smaller mean step lengths (table 1, fig. 7). A habituation effect was revealed by the significant main repetition effect (F(1,25) = 11.75, p = .002) and faster gait velocity on the way back (factor direction: F(1,25) = 31.31, p<.001), and the tendency in the group × direction interaction (F(1,25) = 8.75, p = .007) showed that susceptibles deviated from the controls more strongly on the way out on the balcony than on the way back. One susceptible participant switched to a different gait pattern with a very slow mean walking speed of 0.2 m/s and was thus excluded from gait parameter analysis. Correlation analysis showed moderate correlations of subjective fear with mean gait parameters; however, the correlations had only a tendency to be significant. In the second walk back the correlations almost completely vanished, as participants with greater subjective fear exhibited similar gait parameters as participants with less subjective fear. The lateral distance from the wall was less in susceptibles with a mean and standard deviation of 46±17 cm, while controls exhibited a mean distance of 60±10 cm (t-test, p = 0.0073).

## Discussion

Susceptibles to fear of heights exhibited a limited visual exploration of the surroundings, particularly the depth, during locomotion on an emergency escape balcony. This behavior was associated with a slow, cautious gait.

### Visual exploration and walking behavior during fear at heights

The main difference between susceptibles and controls when walking at heights was that head movements were significantly reduced in all three planes (yaw, pitch, and roll) in susceptibles, whereas total eye movements, with respect to saccade amplitudes and frequencies, did not significantly differ from those of control subjects. However, there was an anisotropy, with a preference for the vertical as opposed to the horizontal direction of saccades. Both effects - reduced head movements in all planes and less frequent horizontal eye movements - led to a smaller total area of visual exploration. Exploration was directed mainly straight ahead and vertically toward the ground in the direction of locomotion. This behavior can be best illustrated by comparing the head movements (fig. 2–4), the eye movement histograms (fig. 5), and the resulting gaze-in-space (fig. 6). Susceptibles tried to avoid looking into the abyss as showed earlier when exposed to height while standing still [2]. This may be part of a strategy to alleviate fear at heights by facing the ground in front of the feet or the handrail of the balcony, which subjectively provides the possibility of physical balance support in case of an impending fall. Individual strategies vary; some subjects focus their gaze on the handrail, some focus on the end goal of the locomotion, while others keep their gaze directed to the closed side of the balcony. Some subjects even kept their arms flexed while walking so as to be prepared to grasp the handrail. Others tried to walk close to the wall rather than in the middle of the balcony.

Quantitative measures of gait parameters demonstrate that susceptibles walk slower than controls; the mean velocity for all four walks (0.90 m/s vs. 1.14 m/s) was significantly reduced. This is reflected in the mean step frequency (1.51 Hz vs. 1.77 Hz) and the mean stride length (0.59 m vs. 0.64 m). Earlier human laboratory experiments also revealed a reduction in velocity, stride length and step frequency in healthy subjects walking on a narrow walkway at a height of 3.5 m compared to ground level [9]. Since these values were still reduced when the visual distance to the surround was artificially decreased with sheets, higher cognitive mechanisms (“danger system”) were thought to drive it: knowledge of the danger was the main influence. Although there was no objective risk of falling on the balcony, the cautious gait pattern found on the escape balcony may also be a result of anxiety. The reduction of gait velocity seems to correlate with subjective fear (table 2). This is compatible with the differential effects of exposure to heights in susceptibles and non-susceptibles to fear of heights. It is well acknowledged that anxiety-related processes affect postural control, e.g., in patients with primary and secondary anxiety disorders [7].

Our measurement technique could only capture the mean values of gait parameters. Gait variability is associated with the risk of falling [16]. Thus, quantitative measurements and the analysis of gait variability and dynamic parameters must still be done in order to further investigate the gait alteration and detect possible implications for the risk of falls during visual exposure to depth.

The locomotion of susceptibles when exposed to height is clinically best described as a slow and cautious, broad-based gait with small steps. Moreover, susceptibles appeared to walk with flat-footed contact and less dynamic vertical oscillations of body and head. Even in those participants with little fear, the holistic inspection of their gait pattern allowed a surprisingly accurate differentiation of susceptibles and non-susceptibles. This gait pattern does not appear to be specific, but is similar to that of others, like the cautious gait observed in children and adults with visual deprivation [17], [18]. In the first clinical description of acrophobia in 1889 [19], the physician Dr. Verga described his own condition: “walking in high places … is getting more and more difficult”. Recent interviews on the quality of life of patients suffering from acrophobia disclosed that subjective imbalance was often characterized as if having heavy or stunned legs: “when it gets really bad, then I can’t even lift my foot. It’s like my feet are glued to the ground.” [20].

### Differential effects of fear of heights on visual control of stance and gait

Vision contributes to multisensory balance control during upright stance [21] as well as during locomotion [22], but it also assists other tasks like navigation and obstacle avoidance. This is reflected in the finding that gaze behavior during locomotion is similar to that exhibited during upright stance.

The area explored visually by susceptible subjects during upright stance is severely reduced, and gaze is preferably directed towards the horizon [2]. The current data collected during locomotion correspond to those during stance, in that the visually explored area was also reduced. Gaze into the depth was also avoided during upright stance. However, exploration of the surround was not directed mostly towards the horizon and saccades were not horizontally oriented along the horizon. The preferred gaze targets were the ground nearby in the heading direction, the handrail, and the goal of the walking path. Saccadic eye movements in the vertical direction were more frequent during locomotion (fig. 5) than in upright stance [2]. Control subjects also exhibited this anisotropy of saccade direction, but it was more pronounced in susceptibles. Thus, the difference is due to the dissimilar view when walking along the balcony. The effect is stronger in susceptibles because they are avoiding the threat that they perceive elsewhere in the visual scene, namely the abyss.

Visual exploration is achieved through the coordination of eye and head movements. During upright stance, both eye and head movements were reduced in susceptibles compared to controls [2]. Our findings during locomotion of susceptibles revealed a substantial and significant reduction in head movements; however, eye movements were not significantly reduced. First, we will discuss the reduction in head movements. The cause of the diminished head movements might be an anxiety-driven stiffening reaction. In a recent study subjects with fear of heights exhibited increased neck muscle co-contraction during exposure to a visual cliff [23]. The link between anxiety and head movement reduction is supported by the significant and strong correlations of subjective fear with reduction in head movements. An additional explanation is given by the findings of Hüweler et al. [12]. They noted that conflicting visual-vestibular sensations evoke anxiety in susceptibles to fear of heights. We believe that a reduction in head movements is meant to minimize visual as well as vestibular stimulation, and therefore to alleviate anxiety. This anxiety-related stiffening did not involve eye movements. With the head kept still, the eyes still explore the surround in order to detect any obstacles, to visually stabilize balance during locomotion, and to check the position of the handrail in case of an impending fall.

When walking on the balcony, visual flow is asymmetric because of the greater distance to the ground on the open side of the balcony. An asymmetric visual flow field has been shown to slow down self-generated gait velocity [24], and conflicting visual flows are known to cause distressing subjective instability in susceptibles to fear of heights [12]. Interestingly, no susceptible subject completely turned his/her head towards the wall, which would have permitted a total avoidance of visual exposure to depth. When the head faces straight ahead, the depth remains a part of the peripheral visual field. Visual control of locomotion has two requirements: to determine the heading direction [25], [26] and to provide stationary contrasts within the visual field for postural stability [27]. Eyes, head, and body are always directed toward the intended path [28].

Thus, restricted visual exploration, particularly avoiding a glance into the depth, is a behavior found in both states: upright stance and walking of subjects susceptible to fear of heights. The different findings for the preferred gaze-in-space (horizontal during stance, vertical during locomotion) are task-dependent and essential for navigation and balance control during locomotion.
